# RNHL1 phase separation coordinates ethylene and gibberellin signaling to regulate wheat plant height

**DOI:** 10.1093/plcell/koag092

**Published:** 2026-03-26

**Authors:** Hongwei Jing

**Affiliations:** Assistant Features Editor, the Plant Cell, American Society of Plant Biologists; Department of Horticultural Science, North Carolina State University, Raleigh, NC 27695, United States

Wheat (*Triticum aestivum*) is a major cereal crop. In the mid-20th century, as part of the Green Revolution, semi-dwarf, high-yielding wheat varieties were identified. The semi-dwarf growth habit is characterized by reduced stem elongation, which increases lodging resistance and reallocates nutrients toward reproductive development, thereby enhancing grain number per spike. The semi-dwarf phenotype is primarily governed by reduced height (*Rht*) genes (Peng et al. 1999). The widely adopted *Rht-B1b* and *Rht-D1b* alleles encode N-terminally truncated DELLA proteins that constitutively repress gibberellin (GA) signaling (Achard et al. 2006). These mutant DELLA proteins exhibit reduced sensitivity to GA-induced proteasomal degradation, resulting in sustained repression of GA-responsive transcriptional activation and consequent restriction of stem elongation (Achard et al. 2006). Recently, *Rht8* was found to encode a protein called Ribonuclease H-Like 1 (RNHL1) that includes a zinc finger DNA-binding motif and an RNase H-like domain (Chai et al. 2022; Xiong et al. 2022), but as yet its mechanism of action has not been clear.

In a recent study, **Chaoqun Dong and colleagues** (Dong et al. 2026) discovered a transcriptional pathway governed by the liquid-liquid phase separation (LLPS) of RNHL1 that regulates plant height in wheat (Fig. 1). By analyzing *RNHL1* knockout and overexpression lines, the authors established that RNHL1 is a critical regulator of plant height and internode elongation. Using traditional yeast 2-hybrid (Y2H) assay, they identified the ETHYLENE-INSENSITIVE3 (EIN3)-LIKE transcription factor (TaEIL1) as an interacting partner of RNHL1. This direct physical interaction was further validated by a suite of protein-protein interaction assays, including firefly luciferase complementation imaging (LCI), bimolecular fluorescence complementation, in vitro pull-down, and in vivo co-immunoprecipitation. Interestingly, fluorescence co-localization analysis showed that RNHL1 and TaEIL1 coassembled into distinct nuclear puncta with signal overlap, a characteristic suggestive of potential LLPS behavior. Furthermore, analysis of intrinsically disordered regions (IDRs) combined with fluorescence recovery after photobleaching assay suggested that both RNHL1 and TaEIL1 possess the capacity to form condensates. Domain mapping revealed that the IDR1 (amino acids 1 to 56) and IDR3 (amino acids 199 to 264) regions of RNHL1 are essential for its phase separation and for its interaction with TaEIL1. Phenotypic characterization further demonstrated that TaEIL1 itself is critical for normal internode elongation and plant height determination in wheat. Collectively, these genetic, biochemical, and phenotypic data support a cooperative role for RNHL1 and TaEIL1 in regulating wheat plant height.

**Figure 1:**
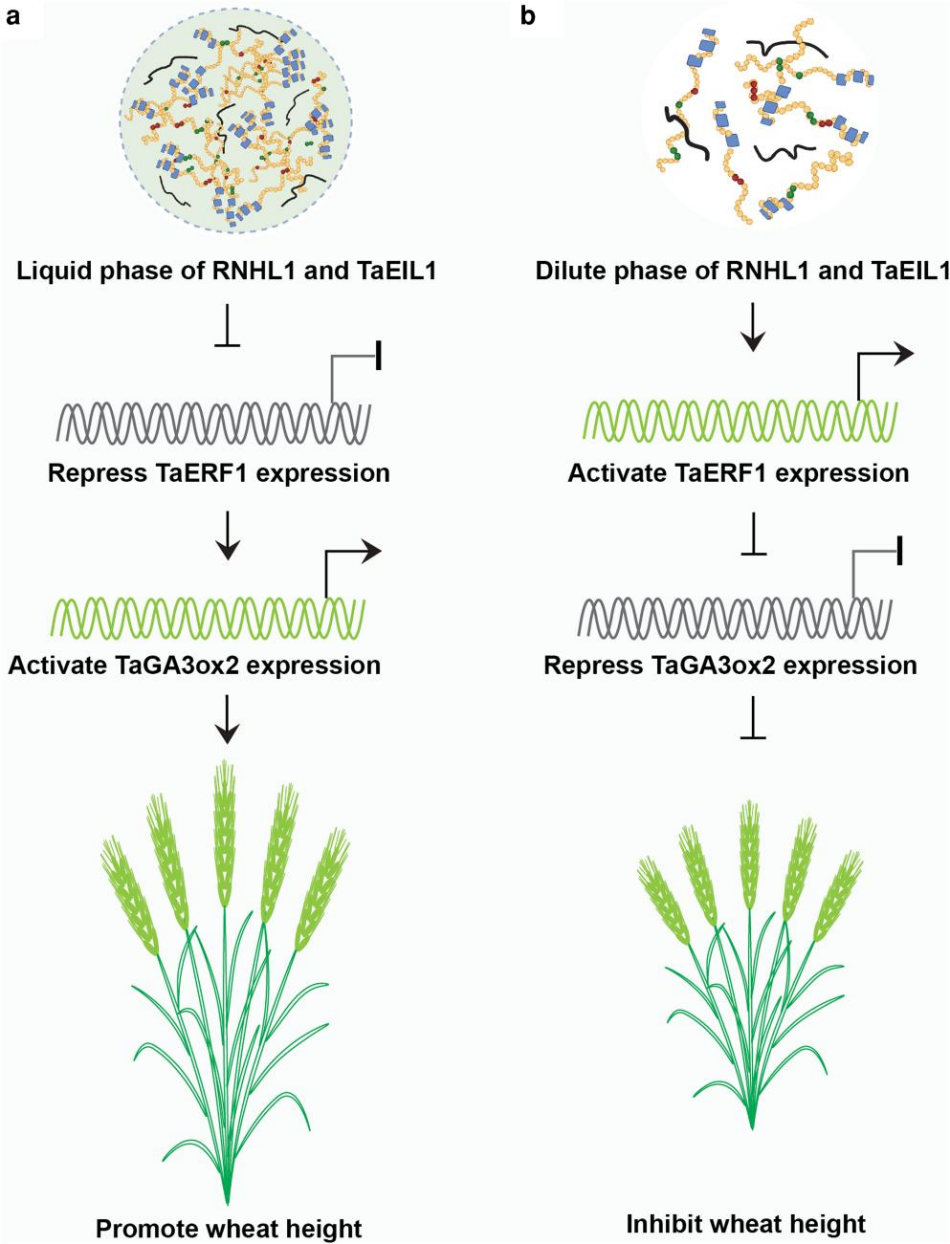
Liquid-liquid phase separation of *Rht8*-derived RNHL1 coordinates ethylene and GA signaling to regulate wheat plant height. a) Liquid-liquid phase separation of Rht8-derived RNHL1 represses *TaERF1*, a transcriptional repressor of the GA biosynthesis gene *TaGA3ox2*. This de-repression elevates GA production, thereby promoting plant growth and height in wild-type wheat. b) In the absence of a functional RNHL1-TaEIL1, which remains in a dilute phase without forming condensates, *TaERF1* is upregulated and directly represses *TaGA3ox2* expression. This leads to reduced GA levels, inhibiting wheat growth and height. Figure credit: H. Jing.

To further elucidate the gene regulatory networks involving *RNHL1* and *TaEIL1*, the authors performed RNA-seq analysis on spike tissues from *rnhl1* and *eil1* mutants. Gene ontology (GO) enrichment and reverse transcription quantitative PCR analyses revealed that genes involved in ethylene and GA signaling pathways are key components of the network modulating plant height and spike length. Specifically, *Ethylene Response Factor 1* (*TaERF1*) was upregulated, while *Gibberellin 3-beta dioxygenase 2* (*TaGA3ox2*), which encodes a GA biosynthetic enzyme, was downregulated. Combined bioinformatic and biochemical assays demonstrated that both RNHL1 and TaEIL1 directly bind to the 5′ untranslated region (5′ UTR) of *TaERF1*, with TaEIL1 additionally targeting its promoter region. Transient expression assays using GFP and firefly luciferase (LUC) reporters showed that RNHL1 and TaEIL1 act synergistically to repress *TaERF1* expression. The synergy of this repression requires both the specific binding of TaEIL1 to the *TaERF1* promoter and the intrinsic phase-separation capacity of RNHL1, suggesting that their physical association and functional interplay are critical for transcriptional control. Phenotypic and biochemical evidence further revealed that TaERF1 directly represses *TaGA3ox2* expression, suppressing GA biosynthesis and consequently reducing plant height. Collectively, these results uncover an RNHL1-TaEIL1-TaERF1-TaGA3ox2 regulatory cascade that mechanistically links ethylene signaling to GA biosynthesis, thereby modulating wheat plant architecture.

Taken together, Dong and colleagues (2026) demonstrate that Rht8-derived RNHL1 mediates crosstalk between ethylene and GA signaling pathways to regulate wheat internode elongation (Fig. 1). Despite these advances, several important mechanistic questions remain unresolved. Both RNHL1 and TaEIL1 exhibit liquid-liquid phase separation properties; however, the molecular determinants and physiological conditions that promote condensate formation remain unclear. For example, what specific structural features or environmental cues drive their assembly into phase-separated complexes? Moreover, how does the RNase H domain contribute to LLPS behavior and condensate stability? Given that LLPS is highly dynamic and context dependent, it will be important to identify the temporal and environmental signals that trigger condensate formation and enable association with the 5′ UTR of *TaERF1*. Understanding when and under what conditions these condensates engage their target will be essential for clarifying their regulatory function. In addition, the RNHL1-TaEIL1-TaERF1-TaGA3ox2 regulatory module ultimately modulates wheat plant architecture through changes in TaGA3ox2 protein abundance. Quantitative analysis of TaGA3ox2 protein levels in *rnhl1 eil1* double mutants and *rnhl1 eil1 erf1* triple mutants would further define the genetic relationships within this pathway. Together, such studies will refine our mechanistic understanding and enhance the potential application of this regulatory module in wheat improvement.

## Recent related articles in *The Plant Cell*:

- Liu and coauthors (Liu et al. 2025) identified a missense mutation in the wheat domestication gene *Q*, which enhances Q protein stability. This enables Q protein to simultaneously repress GA biosynthesis by inhibiting *TaGA3ox2* expression and disrupt GA signaling, thereby modulating plant height and spike length.
- Wang and colleagues (Wang et al. 2025) performed a large-scale population transcriptome and phenotype analysis of 406 wheat accessions, revealing that the Green Revolution allele *Rht-D1b* confers a larger seedling root system by increasing cell length and meristem size, uncovering a previously overlooked benefit of this allele in modern wheat cultivars.
- Hu and colleagues (Hu et al. 2024) identified a Myb73-GDPD2-GA2ox1 transcriptional regulatory module in which GmMyb73 represses *GmGDPD2* expression, enabling its encoded protein to interact with GmGA2ox1 and modulate auxin and GA signaling, thereby enhancing root architecture and phosphate efficiency to confer low-phosphate tolerance in soybean.
- He and colleagues (He et al. 2024) used map-based cloning of a semi-dwarf rice mutant to reveal that OsKANADI1 represses *OsYABBY5* expression to attenuate its activation of the GA catabolism gene *OsGA2ox6*, thereby modulating bioactive GA levels and plant height through a regulatory feedback mechanism.

## Data Availability

No new data were generated or analysed in support of this research.

## Dive Curated Terms

The following phenotypic, genotypic, and functional terms are of significance to the work described in this paper:

EIL1 Gramene: AT2G27050
EIL1 Araport: AT2G27050
